# Chronic Ketamine Exposure Causes White Matter Microstructural Abnormalities in Adolescent Cynomolgus Monkeys

**DOI:** 10.3389/fnins.2017.00285

**Published:** 2017-05-19

**Authors:** Qi Li, Lin Shi, Gang Lu, Hong-Luan Yu, Fu-Ki Yeung, Nai-Kei Wong, Lin Sun, Kai Liu, David Yew, Fang Pan, De-Feng Wang, Pak C. Sham

**Affiliations:** ^1^Department of Psychiatry, The University of Hong KongHong Kong, Hong Kong; ^2^State Key Laboratory for Cognitive and Brain Sciences, The University of Hong KongHong Kong, Hong Kong; ^3^The University of Hong Kong Shenzhen Institute of Research and Innovation (HKU-SIRI), The University of Hong KongHong Kong, Hong Kong; ^4^Department of Medicine and Therapeutics, Chinese University of Hong KongHong Kong, Hong Kong; ^5^Chow Yuk Ho Center of Innovative Technology for Medicine, Chinese University of Hong KongHong Kong, Hong Kong; ^6^School of Biomedical Sciences, Chinese University of Hong KongHong Kong, Hong Kong; ^7^Department of Psychology, Qilu Hospital of Shandong UniversityJinan, China; ^8^Research Center for Medical Image Computing, Department of Imaging and Interventional Radiology, Chinese University of Hong KongHong Kong, Hong Kong; ^9^Chemical Biology Laboratory for Infectious Diseases, Shenzhen Institute of Hepatology, The Third People's Hospital of ShenzhenShenzhen, China; ^10^Department of Psychology, Weifang Medical UniversityWeifang, China; ^11^School of Chinese Medicine, Chinese University of Hong KongHong Kong, Hong Kong; ^12^Department of Medical Psychology, Shandong University School of MedicineJinan, China; ^13^Genome Research Centre, The University of Hong KongHong Kong, Hong Kong

**Keywords:** ketamine, diffusion tensor imaging (DTI), thalamus, frontal gyrus, cynomologus monkeys

## Abstract

Acute and repeated exposures to ketamine mimic aspects of positive, negative, and cognitive symptoms of schizophrenia in humans. Recent studies by our group and others have shown that chronicity of ketamine use may be a key element for establishing a more valid model of cognitive symptoms of schizophrenia. However, current understanding on the long-term consequences of ketamine exposure on brain circuits has remained incomplete, particularly with regard to microstructural changes of white matter tracts that underpin the neuropathology of schizophrenia. Thus, the present study aimed to expand on previous investigations by examining causal effects of repeated ketamine exposure on white matter integrity in a non-human primate model. Ketamine or saline (control) was administered intravenously for 3 months to male adolescent cynomolgus monkeys (*n* = 5/group). Diffusion tensor imaging (DTI) experiments were performed and tract-based spatial statistics (TBSS) was used for data analysis. Fractional anisotropy (FA) was quantified across the whole brain. Profoundly reduced FA on the right side of sagittal striatum, posterior thalamic radiation (PTR), retrolenticular limb of the internal capsule (RLIC) and superior longitudinal fasciculus (SLF), and on the left side of PTR, middle temporal gyrus and inferior frontal gyrus were observed in the ketamine group compared to controls. Diminished white matter integrity found in either fronto-thalamo-temporal or striato-thalamic connections with tracts including the SLF, PTR, and RLIC lends support to similar findings from DTI studies on schizophrenia in humans. This study suggests that chronic ketamine exposure is a useful pharmacological paradigm that might provide translational insights into the pathophysiology and treatment of schizophrenia.

## Introduction

Acute and repeated exposures to ketamine, an *N*-methyl-*D*-aspartate (NMDA) receptor antagonist, can be used to model aspects of positive, negative, and cognitive symptoms of schizophrenia in humans (Krystal et al., 1994; Newcomer et al., 1999; Dawson et al., 2013; Stone et al., 2014). A growing body of neuroimaging studies have demonstrated that acute administration of ketamine induces symptoms in healthy individuals comparable to an acute psychotic state, which include delusions (Abel et al., 2003a; Corlett et al., 2013), dissociative sensation (Deakin et al., 2008; De Simoni et al., 2013) and emotion blunting (Abel et al., 2003b; Daumann et al., 2008). However, a number of studies have indicated that acute ketamine administration does not significantly impair cognitive functions such as working memory, verbal fluency, and attention (Honey et al., 2004, 2005; Fu et al., 2005; Daumann et al., 2008). Furthermore, acute ketamine exposure has yielded inconsistent findings on resting-state functional connectivity particularly prefrontal cortex functional connectivity with subcortical brain regions (Scheidegger et al., 2012; Driesen et al., 2013), and these regions play an important role in executive functions (Bonelli and Cummings, 2007). Recently, it has been postulated that the most-reported short-term psychological effects of ketamine dependence were “floating or circling” sensation, while the long-term effects of ketamine dependence were memory impairment, personality changes, and slowed reactions (Curran and Morgan, 2000; Morgan et al., 2004; Fan et al., 2015). Thus, chronic ketamine use may provide a more valid model of cognitive symptoms of schizophrenia. Improved mechanistic characterization of the effects of long-term ketamine exposure on brain circuits is likewise crucial to a better understanding of the etiology of cognitive symptoms of schizophrenia.

Moreover, compelling phenotypic similarity between chronic ketamine use and schizophrenia exists in functional neural activity or white matter alterations, particularly, in light of disconnections involving the prefrontal cortex. In a recent functional magnetic resonance imaging (*f* MRI) study, it was reported that effects of chronic ketamine use on resting-state functional connectivity are coupled to increased activity in the frontal gyrus and decreased activity in the anterior cingulate cortex (ACC; Liao et al., 2012). Our previous study has also demonstrated that repeated exposure to ketamine in non-human primates reduced functional neural activity in the midbrain, posterior cingulate cortex (PCC), and visual cortex, but increased activity in the striatum (Yu et al., 2012). Diffusion tensor imaging (DTI) is an MRI-based neuroimaging technique that provides information about white matter microstructure *in vivo*. Damages in frontal white matter and corpus callosum have been reported in human cocaine addicts (Moeller et al., 2007; Wang et al., 2013). UP until now, only two clinical DTI findings have reported that chronic ketamine exposure disrupts white matter integrity in the frontal cortex (Liao et al., 2010; Edward Roberts et al., 2014). Liao et al. also reported reduced bilateral frontal gray matter volume in chronic ketamine users (Liao et al., 2011). However, exposure to other psychoactive substances of patients with ketamine dependence may confound interpretation of these results. In view of a paucity of knowledge about the long-term consequences of ketamine administration in brain structures, along with ethical concerns and technical difficulties in addressing this issue in humans, investigation on chronic ketamine exposure in preclinical animal models is clearly warranted.

Adolescents are socially vulnerable to drugs of abuse and display susceptibility to the development of drug dependence (Anthony and Petronis, 1995; Chambers et al., 2003). Despite the high prevalence of ketamine use among adolescents and young adults in Hong Kong and internationally (Lankenau and Clatts, 2004; Tang et al., 2015), disproportionately little is known about its impact on the developing brain. Again, it is particularly challenging to evaluate the drug effects on this age group in clinic. Non-human primates represent an excellent animal model because they share high similarity with humans in the pharmacokinetics and metabolism of several drugs and more importantly their prefrontal cortex is evolutionarily closely aligned with human counterparts (Preuss, 1995; Innocenti et al., 2016). Given the well-established role of prefrontal cortex in cognitive symptoms of psychosis, the aim of the present study was to expand on previous DTI investigations by examining potential causal effects of repeated ketamine exposure on white matter integrity and connectivity profiles between prefrontal cortex and subcortical brain regions in an adolescent non-human primate model. We predicted that chronic ketamine exposure perturbs the integrity of prefrontal cortex and its connectivity with subcortical regions.

## Materials and methods

### Animals and ketamine administration

Male adolescent cynomolgus monkeys (*Macaca fascicularis*) were purchased from Yunnan Laboratory Primate Inc. and all experiments were conducted in full compliance with license from the Ethics Committee of Shandong University. The animals were bred from a colony of natural-habitat-reared *M. fascicularis* and were kept at Hongli Animal Center (approval ID for use of non-human primates in this study: SYXK, 20050041) in a room maintained at temperature of 25°C with a 12:12 h light-dark cycle. Monkeys were individually housed in stainless steel cages (90 × 90 × 70 cm, SCXK Su 2003-0006) with water available *ad libitum* and were allowed to have visual and auditory contact with each other. They were fed twice daily with whole grains diet (Military Medical Animal Center of the Chinese Liberation Army, SCXK), supplemented with fresh fruits each day. Rearing procedures had been reviewed and approved by the Animal Care Committee of Shandong University.

Ten monkeys were randomly divided into two groups: Ketamine (1 mg/kg) or saline (control) was administered intravenously for 3 months to male adolescent *M. fascicularis* at 4.12 ± 0.65 (mean ± standard deviation) years old (ketamine group = 5, control group = 5). Based on studies by our group and others (Stoet and Snyder, 2006; Yu et al., 2012; Sun et al., 2014), ketamine dose of 1 mg/kg in an injection volume of 1 mL in saline was chosen and freshly prepared on the day of injection. Ketamine was given daily intravenously via arm vein under mild physical constraints for 13 weeks (i.e., ~3 months), while control monkeys were given sterile saline 1 mL. Ketamine was not given to the monkeys 24 h before the day of DTI scanning. No other pharmacological agents were administered to monkeys throughout the whole experiments. Animal body weights were recorded monthly to monitor the monkeys' well-being.

### Diffusion tensor imaging (DTI)

DTI experiments were performed on a 3-T Signa Magnetic Resonance System (GE Company). Briefly, monkeys were anesthetized by intramuscular injection with ketamine (10 mg/kg) and xylazine hydrochloride (Sumianxin, 0.1 mL/kg; China Institute of Military Veterinary, Academy of Military Medical Sciences, Changchun, China; Feng et al., 2013). Anesthetized animals were immediately transferred to the MRI room. Anesthesia was sustained throughout the whole scanning period with no extra anesthetic doses being required. The monkeys were kept in place with sponges and sand bags and were oriented in a head-forward sphinx position. Respiration rate and body temperature were continuously monitored during the scan. DTI scanning was performed by using an EP/S sequence with the following parameters: repetition time = 6 s; echo time = 89.8 ms; field of view = 14 cm; flip angle = 90°; matrix = 256 × 256; slice thickness = 2.6 mm. Diffusion weighted imaging (b = 1,000 s/mm^2^) was performed in 25 non-collinear directions with 1 non-diffusion weighted image.

### Data analysis

Fractional anisotropy (FA) was derived from diffusion data which quantifies how strongly directional a local tract structure is. In order to localize brain changes, group comparison of FA-value was performed between control group and ketamine group (*p* < 0.005, uncorrected). Tract-based spatial statistics (TBSS; Smith et al., 2006) part of FSL (Smith et al., 2004) was used in data processing. First, the raw diffusion data were preprocessed and corrected to eliminate the effects of head movement and eddy currents by using FMRIB Diffusion Toolbox (FDT). Then, a binary brain mask was created by brain-extracting the no diffusion weighting (b = 0) image by using Brain Extraction Tool (BET; Smith, 2002) and mean FA images were created by fitting a tensor model to the corrected diffusion data with its brain mask. Before group comparison, all FA images were aligned onto the MNI152 standard space by non-linear registration and resliced into 1 × 1 × 1 mm^3^ resolution by TBSS software for later accurate cluster quantification. A mean FA skeleton was created such that it most represents the centers of all tracts common to the group. The voxel-wise group statistical comparison was performed to show which voxels on the mean FA skeleton mask had significant difference between control group and ketamine group. The analysis statistics was based on TBSS with threshold-free cluster enhancement (TFCE) algorithm. TFCE method was adopted with a non-parametric approach of permutation test of 500 permutations (Smith and Nichols, 2009). Results were generated based on significant threshold at uncorrected one-tailed TFCE *p* < 0.005.The quantification of voxel size within a cluster was then performed by using Xjview toolbox (http://www.alivelearn.net/xjview). To locate the actual anatomical regions of those significant clusters in monkey, a diffusion tensor based white matter brain atlas for rhesus macaques was applied (Adluru et al., 2012) onto the results as anatomical underlay.

## Results

### Regions of significant difference in fractional anisotropy (FA) between groups

Group comparison between chronic ketamine administration group and controls revealed seven different anatomical regions, where the ketamine group had FA-values significantly lower than that of the control group. The anatomic location and size of these clusters with peak *t*-values for ketamine group and controls are as shown in Table 1. In this table, statistical analysis found significant FA changes (*p* < 0.005) between ketamine group and controls in the right side of sagittal striatum (SS; *t* = 4.3829), posterior thalamic radiation (PTR, right side, *t* = 3.9214; left side, *t* = 7.1843), middle temporal gyrus white matter (MTG-WM, left side, *t* = 4.3206), inferior frontal gyrus WM (IFG-WM, left side, *t* = 3.8225), retrolenticular limb of the internal capsule (RLIC, right side, *t* = 2.5386), and superior longitudinal fasciculus (SLF, right side, *t* = 4.9845).

**Table 1:** **Regions with significant FA differences between chronic ketamine exposure group and controls**.

**Region of significant clusters**	**Hemisphere**	**Cluster size (voxels)**	**Peak coordinates [*x*, *y*, *z*]**	**Peak *t*-value**
Sagittal striatum (SS)	Right	7	[52, 27, 33]	4.3829
Posterior thalamic radiation (PTR)	Right	27	[54, 23, 35]	3.9214
Posterior thalamic radiation (PTR)	Left	12	[20, 27, 35]	7.1843
Middle temporal gyrus WM (MTG-WM)	Left	4	[15, 37, 37]	4.3206
Inferior frontal gyrus WM (IFG-WM)	Left	3	[23, 61, 43]	3.8225
Retrolenticular limb of the internal capsule (RLIC)	Right	4	[52, 38, 38]	2.5386
Superior longitudinal fasciculus (SLF)	Right	21	[54, 27, 42]	4.9845

*x, y, z are co-ordinates based on the reference DTI monkey brain template FA map (Adluru et al., 2012) with its atlas (http://www.nitrc.org/projects/rmdtitemplate/). z indicates the vertical distance from the lowest axial plane of reference image in mm, x refers to sagittal plane, y refers to coronal plane. The uncorrected p-value of all the voxels in the cluster are p < 0.005. WM, white matter*.

**Figure 1:**
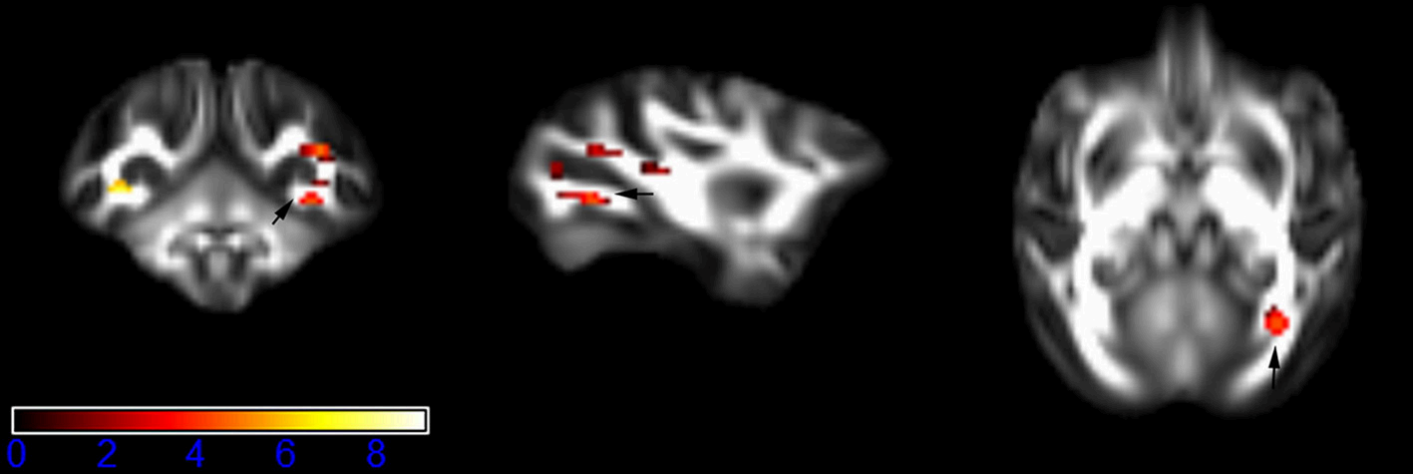
**Areas in sagittal striatum (SS, right side) with reduced fractional anisotropy values in the chronic ketamine exposure group compared to controls at ***p*** < 0.005 (in red; uncorrected)**. The anatomical underlay is DTI-based monkey brain image template (UWRMAC-DTI271) FA map with white matter atlas. Figure from left to right side is coronal view, sagittal view, and axial view of the images, respectively. The color bar represents the *t*-value.

**Figure 2:**
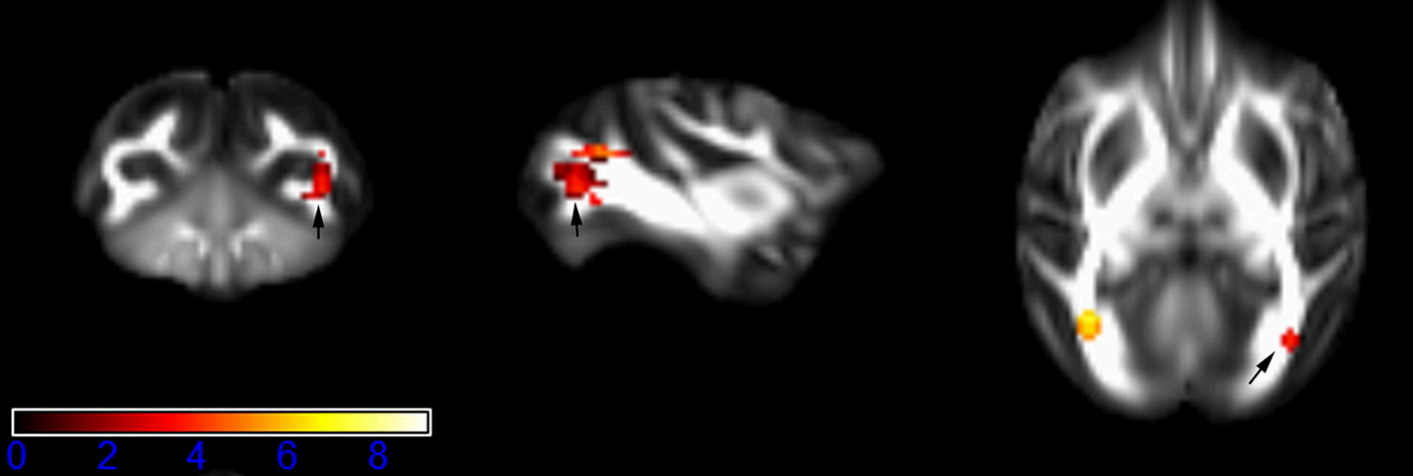
**Areas in posterior thalamic radiation (PTR, right side) with reduced fractional anisotropy values in the chronic ketamine exposure group compared to controls at ***p*** < 0.005 (in red; uncorrected)**. The anatomical underlay is DTI-based monkey brain image template (UWRMAC-DTI271) FA map with white matter atlas. Figure from left to right side is coronal view, sagittal view, and axial view of the images, respectively. The color bar represents the *t*-value.

**Figure 3:**
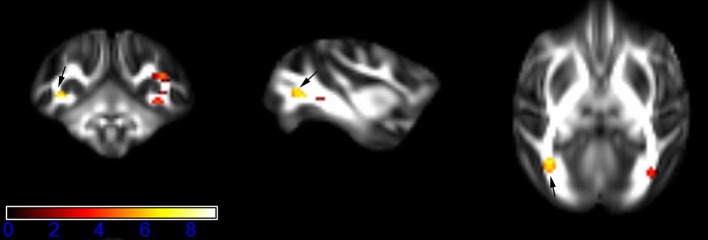
**Areas in posterior thalamic radiation (PTR, left side) with reduced fractional anisotropy values in the chronic ketamine exposure group compared to controls at ***p*** < 0.005 (in red; uncorrected)**. The anatomical underlay is DTI-based monkey brain image template (UWRMAC-DTI271) FA map with white matter atlas. Figure from left to right side is coronal view, sagittal view, and axial view of the images, respectively. The color bar represents the *t*-value.

**Figure 4:**
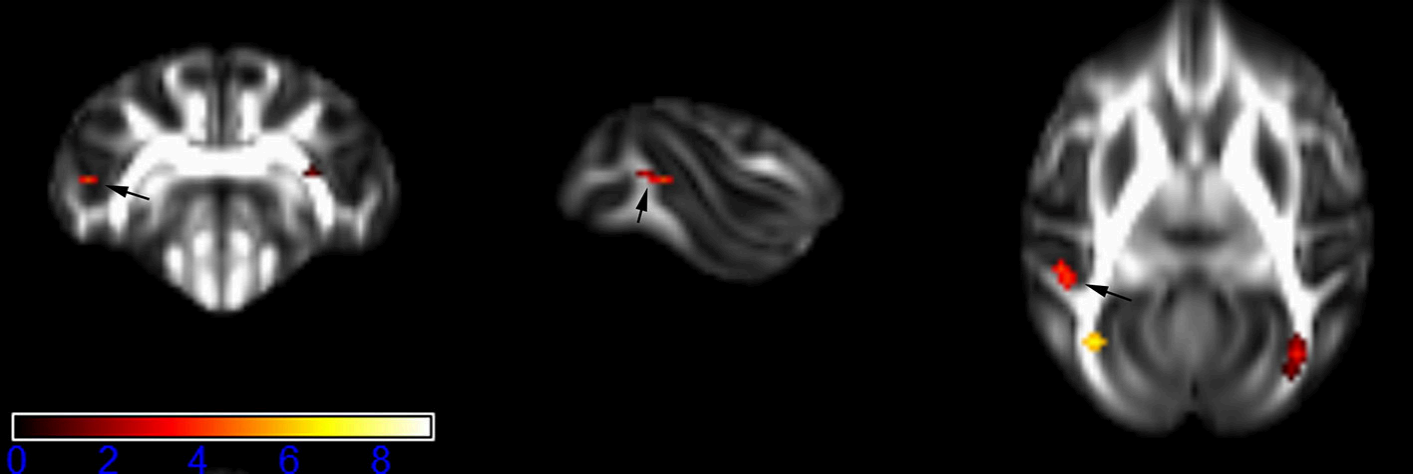
**Areas in middle temporal gyrus WM (MTG-WM, left side) with reduced fractional anisotropy values in the chronic ketamine exposure group compared to controls at ***p*** < 0.005 (in red; uncorrected)**. The anatomical underlay is DTI-based monkey brain image template (UWRMAC-DTI271) FA map with white matter atlas. Figure from left to right side is coronal view, sagittal view, and axial view of the images, respectively. The color bar represents the *t*-value.

**Figure 5:**
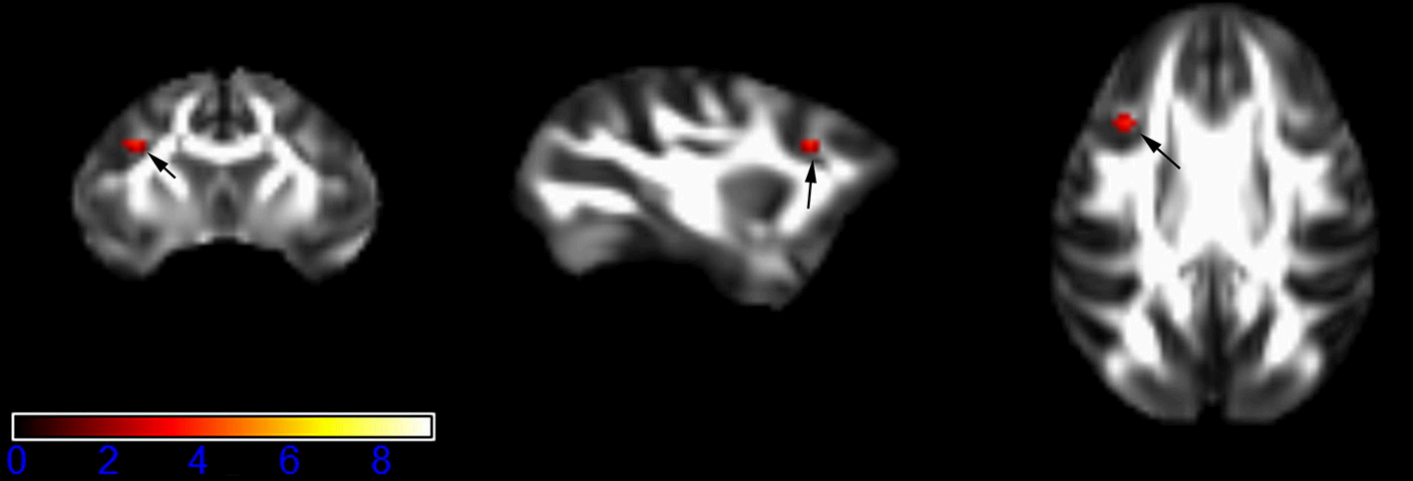
**Areas in inferior frontal gyrus WM (IFG-WM, left side) with reduced fractional anisotropy values in the chronic ketamine exposure group compared to controls at ***p*** < 0.005 (in red; uncorrected)**. The anatomical underlay is DTI-based monkey brain image template (UWRMAC-DTI271) FA map with white matter atlas. Figure from left to right side is coronal view, sagittal view, and axial view of the images, respectively. The color bar represents the *t*-value.

**Figure 6:**
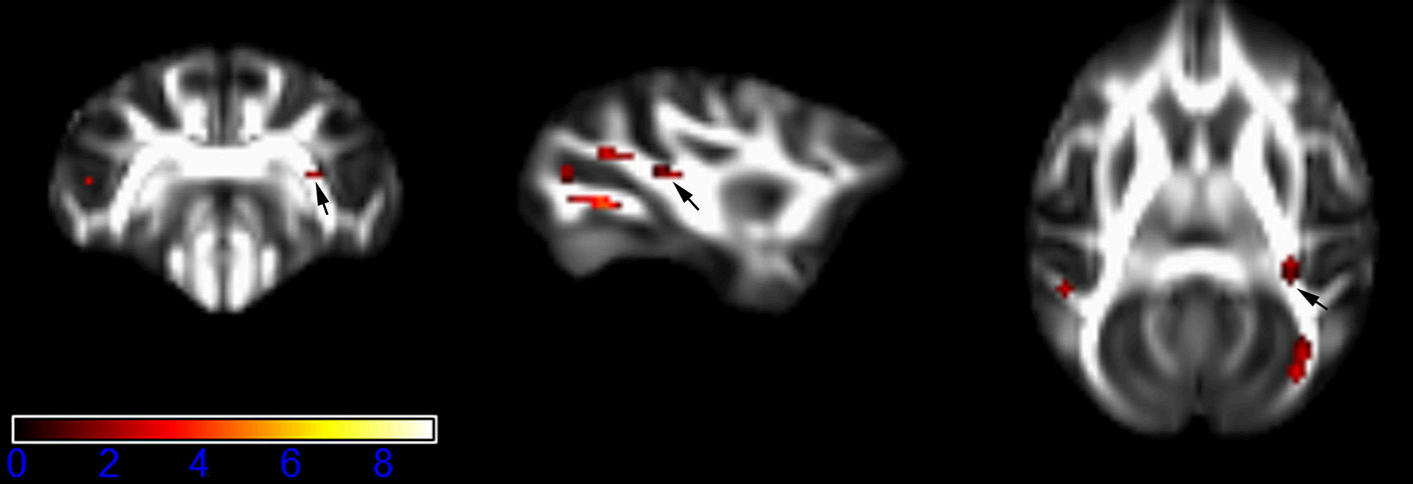
**Areas in retrolenticular limb of the internal capsule (RLIC, right side) with reduced fractional anisotropy values in the chronic ketamine exposure group compared to controls at ***p*** < 0.005 (in red; uncorrected)**. The anatomical underlay is DTI-based monkey brain image template (UWRMAC-DTI271) FA map with white matter atlas. Figure from left to right side is coronal view, sagittal view, and axial view of the images, respectively. The color bar represents the *t*-value.

**Figure 7:**
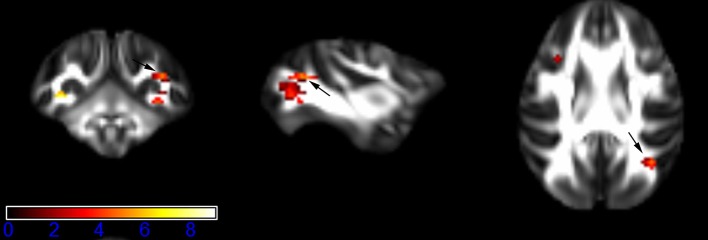
**Areas in superior longitudinal fasciculus (SLF, right side) with reduced fractional anisotropy values in the chronic ketamine exposure group compared to controls at ***p*** < 0.005 (in red; uncorrected)**. The anatomical underlay is DTI-based monkey brain image template (UWRMAC-DTI271) FA map with white matter atlas. Figure from left to right side is coronal view, sagittal view, and axial view of the images, respectively. The color bar represents the *t*-value.

Figure 1 showed spatial distribution of the brain regions indicating a reduction of FA (*p* < 0.005, TFCE-uncorrected) in right SS in the ketamine administration group when compared with controls. In addition, the significant regions were located in the posterior region of the thalamic radiation. Both right and left PTR showed reduced FA (*p* < 0.005) in the ketamine administration group (Figures 2, 3). The RLIC comes from the thalamus and more posteriorly this becomes the optical radiation (Larry et al., 2012). Figure 6 indicated a reduction of FA (*p* < 0.005) in the right RLIC in the ketamine group compared with controls. SLF, the largest association bundles, connects to the cortex of the frontal, parietal, occipital and temporal lobes (Larry et al., 2012). We found reduced FA in the right SLF in ketamine administration group (*p* < 0.005; Figure 7). Consistent with a ground-breaking finding reported by Liao et al. (2010), the present study showed decreased FA (*p* < 0.005) in left side of IFG (Figure 5) and MTG (Figure 4) in chronic ketamine administration group compared with controls. Moreover, our previous functional image study on the same model showed hypofunctions in the PCC and visual cortex (Yu et al., 2012).

There were no cluster areas where the ketamine group had FA-values higher than that of controls.

## Discussion

The present study provides direct experimental evidence that chronic ketamine exposure significantly disrupts white matter microstructures across a number of critical brain circuits. We found that repeated ketamine exposure elicits bidirectional reduced FA of PTR, left side of middle temporal gyrus WM (MTG-WM) and inferior frontal gyrus WM (IFG-WM), and also reduced FA in the right side of sagittal striatum (SS), RLIC, and SLF. More importantly, reduced integrity of fronto-thalamo-temporal or striato-thalamic white matter connectivity observed in this study is consistent with, and extend the previous clinical findings in chronic ketamine users.

Our current DTI findings of abnormality in the IFG-WM and MTG-WM with chronic ketamine exposure in non-human primate are largely consistent with white matter tract patterns observed in previous clinical imaging studies of schizophrenia in humans (Thompson et al., 2001; Kyriakopoulos et al., 2008; Ellison-Wright and Bullmore, 2009). In a comprehensive meta-analysis of DTI studies on schizophrenia, Ellison-Wright et al. (Ellison-Wright and Bullmore, 2009) summarized two locations of FA reductions in the deep white matter of the left frontal and temporal lobes. Disruption of fronto-temporal white matter network may contribute to cognitive deficits in schizophrenia (Kubicki et al., 2007). Even though abnormalities in the white matter have not been found consistently in schizophrenia patients in methodologically varied studies, the frontal white matter seems to be commonly affected. Recently, Zalesky et al. (2011) showed that a fronto-parietal/occipital network may represent the key macro-circuit affected in schizophrenia, while an aberrant network structure of bilateral inferior frontal cortex and temporal has been reported in schizophrenia patients (van den Heuvel et al., 2010). These findings suggest that schizophrenia patients have a less organized brain networks with a reduced central role for the key frontal hub, which results in limited integration of information between brain regions.

Consistent with the view of “hypofrontality” in schizophrenia (Wolkin et al., 1992), a growing number of brain imaging studies have shown that the frontal white matter abnormalities may be as a fundamental change in patients with chronic drug dependence (Nestler, 2005; Wang et al., 2013). Animal studies by our group and others have shown that apoptosis of neuronal cells in the frontal cortex is induced by chronic exposure to ketamine (Zou et al., 2009; Yeung et al., 2010; Sun et al., 2014). To date, however, there have been only two clinical studies examining white matter integrity in chronic ketamine users (Liao et al., 2010; Edward Roberts et al., 2014). Liao et al. (2010) first reported that white matter changes with reduced FA in the bilateral frontal and left temporo-parietal cortices are associated with chronic ketamine use. Later, Edward Roberts et al. (2014) partially replicated the findings reported by Liao et al., which revealed a reduction in axial diffusivity in the right side of prefrontal white matter in chronic ketamine users. They further observed that the connectivity between caudate nucleus and lateral prefrontal cortex pathway was positively associated with dissociative experiences in ketamine users (Edward Roberts et al., 2014). Our findings are also in partial agreement with those studies, as the brain areas of abnormality observed in this study were also located in the left side of frontal lobe (IFG-WM) and temporal lobe (MTG-WM). The absence of an effect of ketamine on right side of frontal lobe in this study may be because a shorter duration of ketamine exposure or the use of only male animals in our experiment. A second consideration is that all the subjects in our study started to receive ketamine during adolescence, which is a stage of development known to involve brain plasticity (Blakemore and Choudhury, 2006). In addition, frontal lobe deficits may well be associated with the ketamine-related memory defects and cognitive symptoms in schizophrenia patients. Our research group have demonstrated that chronic exposure to ketamine impaired working memory in mice, which was correlated with dysfunction of GABA signaling system in the prefrontal cortex (Tan et al., 2011). However, further investigation including studies on working memory symptoms combined with neuroimaging is needed to better understand the neuropathological roles of the frontal lobe in chronic ketamine users.

Both IFG-WM and MTG-WM lie laterally to the SLF (Adluru et al., 2012), the major white matter connection between the prefrontal and parietal/temporal cortices, which are functionally related to verbal working memory performance (Hazlett et al., 2008; Karlsgodt et al., 2008). Edward Roberts et al. (2014) reported reduced axial diffusivity in the right side of SLF in ketamine users. In addition, recent evidence has demonstrated that activities in the right IFG network connected by SLF, especially in the right hemisphere, play prominent roles in corporeal awareness during illusion (Amemiya and Naito, 2016). Patients with first-episode paranoid schizophrenia exhibited reduced FA in the right SLF and right internal capsule (Guo et al., 2012). In line with these findings, decreased FA in the right SLF in our study may implicate fronto-parietal white matter disconnectivity in symptoms associated with chronic ketamine exposure in monkeys.

White matter FA in bilateral PTR, a region encompassing fiber pathways that connects the caudal parts of thalamus with the occipital and parietal lobes, was reduced in monkeys chronically exposed to ketamine. This association tract is of interest because it plays a key role connecting visual and motor processes, and is an integral part of neural network regulating cognitive performance (Cremers et al., 2016). Bilateral FA reduction in the PTR has been reported in patients with schizophrenia (Peters et al., 2010; Melonakos et al., 2011; Melicher et al., 2015). However, Edward Roberts et al. (2014) found impairment only on the right side of PTR in chronic ketamine users. Our results encourage further investigation into the role of this projection in ketamine use.

The sagittal striatum, which includes a large part of both the inferior longitudinal fasciculus and inferior fronto-occipital fasciculus, projects to the frontal and limbic cortices (Adluru et al., 2012). Striatum and its cortical connections are critical in the pathogenesis of the complex cognitive symptoms of schizophrenia (Simpson et al., 2010). There is corroborative evidence from animal models and clinical investigations that sensorimotor gating function is modulated by cortico-striato-thalamic circuitry (Hazlett et al., 2008; Li et al., 2009, 2010). In the present study, compromised white matter tract integrity seen on right side of striato-thalamic connections, specifically tracts including RLIC and PTR, supports white matter alterations in the brain of chronic ketamine users as reported by Edward Roberts et al. (2014). These white matter alterations in the brain may also contribute to the dissociative experiences reported by chronic ketamine users (Edward Roberts et al., 2014). Moreover, reduced FA in RLIC was reported to be associated with negative symptoms in patients with schizophrenia (Arnedo et al., 2015).

## Conclusion

The results of the present study confirm chronic ketamine use during adolescence causes brain damage in areas known to be involved in neurodevelopment during adolescence, in particular, fronto-thalamo-temporal white matter connection (Alcauter et al., 2014; Xiao et al., 2016). Based on clinical findings on chronic ketamine exposure, our study has systematically surveyed major brain regions posited to have reductions in white matter integrity. More importantly, the current observations of reduced white matter in eitherfronto-thalamo-temporal or striato-thalamic networks involving white matter tracts including the SLF, PTR, and RLIC echo findings in human DTI studies on schizophrenia. These results furnish a rational basis for studying chronic ketamine exposure of adolescent brain as a pharmacological paradigm and may yield translational insights into the pathophysiology and treatment of schizophrenia.

## Limitations

Firstly, we acknowledge that the sample size of the current study was modest. Our findings are nonetheless promising, being the first direct evidence for establishing the casual effects of chronic ketamine exposure on microstructure integrity of white matter tracts in adolescent non-human primates. Secondly, we examined only male adolescent monkeys in this study. A decision to dedicate finite experimental resources to males was made because male subjects are generally more vulnerable to drug addiction (DePoy et al., 2016), and there is evidence that males experiencing neurodevelopmental perturbations associated with schizophrenia were inferior to females in memory performance (Goldstein et al., 1998).

## Author contributions

QL, LS (2nd author), GL, DY, FP, DW, and PS contributed to design, acquisition, analysis, and interpretation of data for this work. QL wrote the draft for the manuscript. GL, HY, LS (7th author), and FP performed the experiments. LS (2nd author), FY, KL, QL, and DW prepared all figures and image analysis. NW, GL, HY, LS (2nd author), and PS revised and edited the manuscript. PS approved the final draft of the manuscript. All authors reviewed the manuscript for intellectual content and approved submission.

## Funding

Research reported in this publication was supported by a National Natural Science Foundation of China (NSFC) grant (NO. 81300987).

### Conflict of interest statement

The authors declare that the research was conducted in the absence of any commercial or financial relationships that could be construed as a potential conflict of interest.
